# New ^55^Co-labeled Albumin-Binding Folate Derivatives as Potential PET Agents for Folate Receptor Imaging

**DOI:** 10.3390/ph12040166

**Published:** 2019-11-08

**Authors:** Lauren L. Radford, Solana Fernandez, Rebecca Beacham, Retta El Sayed, Renata Farkas, Martina Benešová, Cristina Müller, Suzanne E. Lapi

**Affiliations:** 1 Department of Radiology, University of Alabama at Birmingham, Birmingham, AL 35233, USA; lauren.radford@curiumpharma.com (L.L.R.); sfernandez@uabmc.edu (S.F.); beachambecca@gmail.com (R.B.); relsay2@emory.edu (R.E.S.); 2 Center for Radiopharmaceutical Sciences ETH-PSI-USZ, Paul Scherrer Institute, 5232 Villigen-PSI, Switzerland; dr.renata.farkas@gmail.com (R.F.); m.benesova@dkfz-heidelberg.de (M.B.); cristina.mueller@psi.ch (C.M.); 3 Department of Chemistry and Applied Biosciences, ETH Zurich, 8093 Zurich, Switzerland; 4 Department of Chemistry, University of Alabama at Birmingham, Birmingham, AL 35205, USA

**Keywords:** folic acid, folate receptors, cobalt-55, PET imaging, albumin binder, ovarian cancer

## Abstract

Overexpression of folate receptors (FRs) on different tumor types (e.g., ovarian, lung) make FRs attractive in vivo targets for directed diagnostic/therapeutic agents. Currently, no diagnostic agent suitable for positron emission tomography (PET) has been adopted for clinical FR imaging. In this work, two ^55^Co-labeled albumin-binding folate derivatives-[^55^Co]Co-cm10 and [^55^Co]Co-rf42-with characteristics suitable for PET imaging have been developed and evaluated. High radiochemical yields (≥95%) and in vitro stabilities (≥93%) were achieved for both compounds, and cell assays demonstrated FR-mediated uptake. Both ^55^Co-labeled folate conjugates demonstrated high tumor uptake of 17% injected activity per gram of tissue (IA/g) at 4 h in biodistribution studies performed in KB tumor-bearing mice. Renal uptake was similar to other albumin-binding folate derivatives, and liver uptake was lower than that of previously reported [^64^Cu]Cu-rf42. Small animal PET/CT images confirmed the biodistribution results and showed the clear delineation of FR-expressing tumors.

## 1. Introduction

The overexpression of the folate receptor (FR) on various cancer types makes it an attractive target for diagnostic radiopharmaceuticals. Several epithelial cancer types, such as ovarian, lung and breast, overexpress the FR alpha isoform (FRα) [1,2]. In the case of ovarian cancer, which is typically not diagnosed until a late stage, studies show that as high as 90% of ovarian carcinomas overexpress FRs relative to healthy tissue, making early detection of FR-expressing tumors highly desirable for improving clinical outcomes [3]. Thus, there is a well-defined need for a high-resolution diagnostic agent capable of reliably monitoring FR expression. Such a diagnostic tool could help inform physicians of a patient’s clinical status and aid in the development of individualized treatment plans. 

Up to now, only two FR-imaging agents have been used in clinical trials, [^99m^Tc]Tc-EC20 and [^111^In]In-DTPA-folate, and only [^99m^Tc]Tc-EC20 is currently used in investigational studies under the trade name [^99m^Tc]Tc-Etarfolatide (Endocyte Inc., West Lafayette, IN, USA) [4,5]. Additionally, these two agents can only be imaged using single photon emission computed tomography (SPECT). To date, no folate-based radiopharmaceuticals capable of utilizing the higher resolution and quantitative information offered by positron emission tomography (PET) have been adopted for clinical use, although there is (very recently) a new ^18^F-labeled agent under investigation in a clinical trial in Europe [6]. Thus, a PET agent for FR-imaging utilizing cobalt-55 (^55^Co, t_1/2_ = 17.5 h, Eβ^+^_avg_ = 0.57 MeV, I = 76%) was developed and studied pre-clinically.

**Figure 1:**
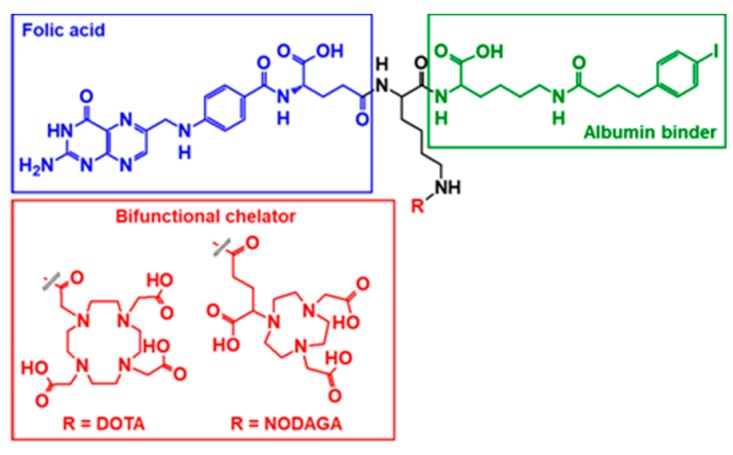
Structures of the albumin-binding folate derivatives cm10 (R = 1,4,7,10-tetraazacyclododecane-1,4,7,10-tetraacetic acid (DOTA)) and rf42 (R = 1,4,7-triazacyclononane, 1-glutaric acid-4,7-acetic acid (NODAGA)). In addition to a bifunctional chelator, each bioconjugate consists of a folic acid molecule for FR-targeting and a *p*-iodophenyl-based albumin-binding entity.

The primary challenge for FR-targeted radiopharmaceuticals is the significant non-tumor uptake in the kidneys, due to the natural expression of FRs in the proximal tubules, resulting in a higher dose burden to the kidneys and negatively impacting tumor-to-background ratios [7,8,9]. Reduction of renal uptake has been achieved by pre-injection with an anti-folate agent (e.g., pemetrexed), conjugation of an albumin-binding entity to the radiopharmaceutical or a combination of both [10,11,12,13,14,15]. The properties of albumin-binding agents can be leveraged to decrease undesired renal uptake of bioconjugates by increasing blood circulation time and reducing clearance rates, thereby enhancing tumor-uptake [16]. Two of these derivatives-cm10 and rf42 (Figure 1)-contain the bifunctional chelating agents 1,4,7,10-tetraazacyclododecane-1,4,7,10-tetraacetic acid (DOTA) and 1,4,7-triazacyclononane, 1-glutaric acid-4,7-acetic acid (NODAGA), respectively, and each has been previously radiolabeled with positron-emitters copper-64 (^64^Cu, t_1/2_ = 12.7 h, Eβ^+^_avg_ = 0.278 MeV, I = 17.6%) and gallium-68 (^68^Ga, t_1/2_ = 1.1 h, Eβ^+^_avg_ = 0.829 MeV, I = 88.9%) and evaluated pre-clinically [14]. 

It has been shown that the pharmacokinetic profile of a small molecule can be altered by making small molecular substitutions (e.g., chelator, complexed metal) [14,17,18,19,20]. In this case, the choice of ^55^Co lies in part to its similarity to ^64^Cu (e.g., oxidation state, half-life), but more importantly, in the interesting advantages it offers. Cobalt(II) is not subject to in vivo reduction and transchelation by metalloproteins in the blood—such as occurs with Cu(II) and transcuprein-and consequently ^55^Co-labeled compounds can offer reduced liver uptake in comparison to their ^64^Cu-labeled counterparts, with free ^55^Co behaving as a calcium mimetic [21]. Additionally, its positron branching ratio is higher than that of ^64^Cu, requiring less injected activity and/or less scanner time to obtain an image of comparable quality. In a recent study, the ability to inject less ^55^Co lead to a similar effective total dose for a ^55^Co-labeled agent in comparison to that of the ^64^Cu-labeled analog, while also maintaining higher imaging contrast and higher tumor-to-background ratios. [22] No ^55^Co-labeled radiofolates have been reported prior to this work, and so both [^55^Co]Co-cm10 and [^55^Co]Co-rf42 were synthesized and evaluated to determine which chelator (DOTA or NODAGA) might offer better properties for ^55^Co-binding.

## 2. Results and Discussion

### 2.1. Radiolabeling

**Figure 2:**
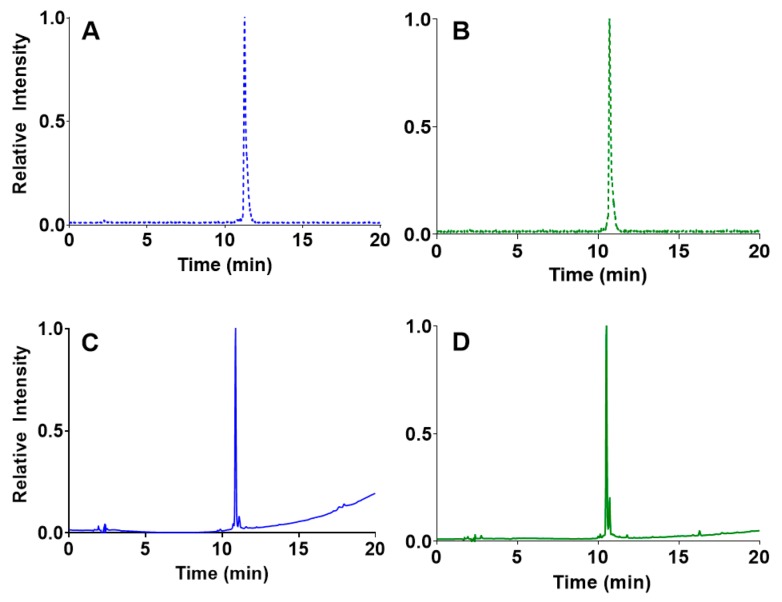
HPLC chromatograms of the radiofolates (**A**) [^55^Co]Co-cm10 and (**B**) [^55^Co]Co-rf42 (NaI detector). The non-radioactive precursors (**C**) cm10 and (**D**) rf42 are shown below their radiolabeled counterparts (UV detector, 254 nm).

Heating [^55^Co]CoCl_2_ in the presence of either cm10 or rf42 at pH 5.5–6.5 resulted in the formation [^55^Co]Co-cm10 or [^55^Co]Co-rf42, respectively. HPLC analyses of the radiofolates showed an increase in retention time from 2.2 min for free [^55^Co]CoCl_2_ to 11.3 min for [^55^Co]Co-cm10 (Figure 2A) and 10.7 min for [^55^Co]Co-rf42 (Figure 2B). The retention times of the radiofolates closely matched the retention times of 10.9 min and 10.5 min of the non-radiolabeled precursors cm10 (Figure 2C) and rf42 (Figure 2D), respectively. Radio-TLC was performed using aluminum-backed silica plates developed in a citric acid buffer (pH 4.6), in which free [^55^Co]CoCl_2_ moved to the solvent front and the radiolabeled ^55^Co-folate compounds had R_f_ values of 0.2–0.3. Radiochemical yields for both compounds were consistently > 95% with molar activity ranging from 300.0–1328.3 MBq/µmol (8.1–35.9 mCi/µmol). The ^55^Co-labeled folates were used for subsequent experiments without further purification. Production of higher quantities of ^55^Co would likely improve the effective specific activity of the [^55^Co]CoCl_2_ starting material, and therefore, improve the molar activity of the final radiolabeled compounds.

### 2.2. In Vitro Stability Determination

Stability of the ^55^Co-labeled folates was monitored over a period of 24 h in phosphate buffered saline (PBS) and mouse serum, to monitor the stability of a dose prior to injection (for potential shipment to a PET center) and match the longest biodistribution time point, respectively. The percentage of the intact complex was monitored at 1 h, 4 h, and 24 h via radio-TLC (PBS) or HPLC (mouse serum). The results indicate that [^55^Co]Co-cm10 and [^55^Co]Co-rf42 exhibit similar stability profiles and remain highly stable over a period of 24 h (Table 1 and Table 2). In PBS, both radioconjugates retained 93% or greater stability at 24 h. For mouse serum stability, the HPLC chromatograms (Figures S1 and S2) showed the ingrowth of new peaks at 11.0 min ([^55^Co]Co-cm10) and 10.9 min ([^55^Co]Co-rf42) over the course of 24 h, presumably due to interaction of the radiofolates with serum proteins. As a control, one group of replicate serum tubes contained only [^55^Co]CoCl_2._ Analysis of the control group showed a single peak at 2.2 min, and so it is unlikely that the unidentified peaks in the HPLC chromatograms of the radiofolates are due to loss of ^55^Co from the complex. Therefore, the peak corresponding to free [^55^Co]CoCl_2_ (t_R_ = 2.2 min) was used to determine the overall serum stability of the ^55^Co-labeled chelates, which was approximately 95% for each compound at 24 h. 

### 2.3. Cell Binding and Internalization

**Figure 3:**
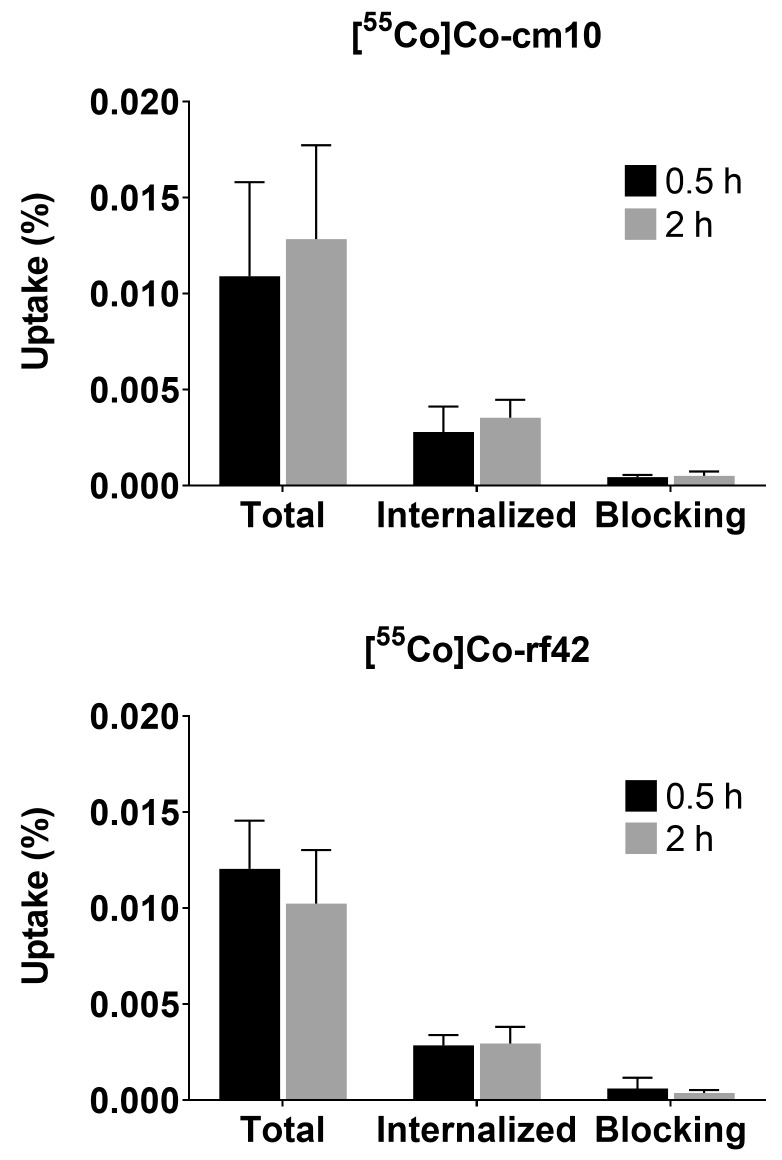
Uptake and internalization of (top) [^55^Co]Co-cm10 and (bottom) [^55^Co]Co-rf42 in KB tumor cells. Blocking was performed by co-incubation with 0.7 mM of folic acid.

Cellular assays were performed using the human cervical cancer KB cell line, which was cultured in folate-deficient cell media prior to use. The ^55^Co-labeled folates were incubated with FR-expressing KB cells to determine total uptake (i.e., all cell-associated activity, both surface-bound and internalized) and the internalized fraction. To determine whether uptake was receptor-mediated, some cells were co-incubated with folic acid, which binds to FRs with high affinity to act as a blocking agent. In comparison to the previously reported ^64^Cu- and ^68^Ga-labeled agents, the overall uptake was significantly lower for the ^55^Co-labeled radiofolates (0.011–0.012%), due to the presence of excess nonradioactive precursor (1.1 μmol) versus the previous studies (1.4–1.5 nmol) [14]. Nonetheless, receptor-mediated uptake was demonstrated as the addition of a blocking agent resulted in a statistically significant reduction in uptake. The uptake profiles for both of the tested radiofolates were similar to each other and more similar to the previously reported [^68^Ga]Ga-rf42 complex, in contrast to [^64^Cu]Cu-rf42 [14]. In the case of [^55^Co]Co-cm10, 26% of the total uptake was internalized with 95% of the uptake blocked by co-incubation of the cells with 0.7 mM of folic acid (Figure 3). Similarly, 24% of the total [^55^Co]Co-rf42 uptake was internalized into the cell, while 97% of the total uptake was blocked upon co-incubation with folic acid (Figure 3), thus, demonstrating receptor-mediated uptake.

### 2.4. In Vivo Biodistribution and PET Imaging

**Figure 4:**
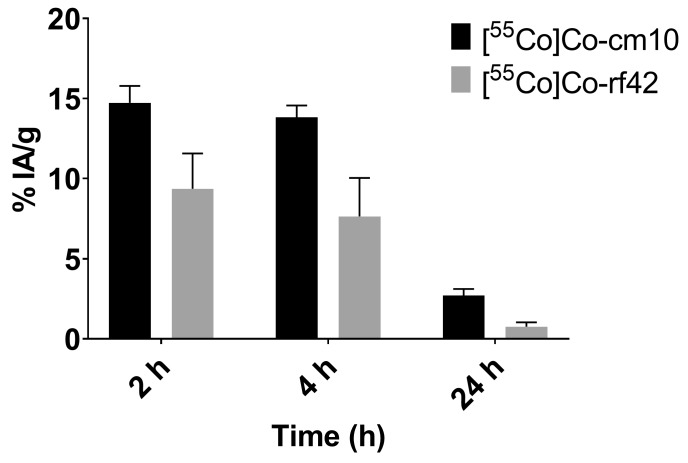
Blood radioactivity values for [^55^Co]Co-cm10 and [^55^Co]Co-rf42 in KB-tumor bearing mice at 2 h, 4 h and 24 h post-injection.

**Figure 5:**
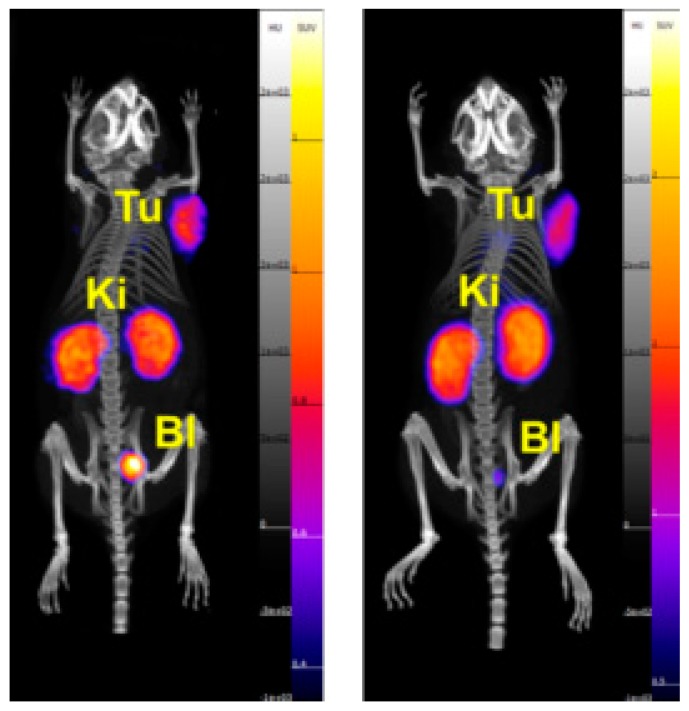
Images of KB tumor-bearing mice injected with (left) [^55^Co]Co-cm10 and (right) [^55^Co]Co-rf42 at 4 h post-injection. Shown as maximal intensity projections. (Tu = tumor, Ki = kidney, Bl = bladder).

The in vivo behavior of each ^55^Co-labeled folate was evaluated in KB-tumor bearing mice via biodistribution studies and PET/CT imaging. The biodistribution data are presented in Table 3 and Table 4, and are reported as the percent of injected activity per gram of tissue (% IA/g). Statistically significant differences in renal uptake between the two ^55^Co-labeled compounds were observed. At 4 h post-injection, the radiofolates had uptake values in the kidneys of 36% ± 7% IA/g for [^55^Co]Co-cm10 and 53% ± 12% IA/g for [^55^Co]Co-rf42. A similar trend was reported for ^64^Cu-labeled PSMA agents, where the NODAGA-functionalized agent exhibited higher kidney retention than the DOTA-functionalized bioconjugate [23]. Lower renal uptake for [^55^Co]Co-cm10 may in part be due to slightly higher lipophilicity (supported by its larger molecular weight and a longer HPLC-retention time) in comparison to [^55^Co]Co-rf42. This was also evidenced in the blood profiles of [^55^Co]Co-cm10 and [^55^Co]-rf42 (Figure 4), which showed higher levels of [^55^Co]Co-cm10 at each evaluated time point. Despite the differences in blood retention, tumor uptake for both radiofolates was similar with values of 17% ± 2% IA/g for [^55^Co]Co-cm10 and 17% ± 4% IA/g for [^55^Co]Co-rf42 at 4 h post-injection. Co-injection of folic acid as a blocking agent showed significant reduction of uptake in FR-expressing tissues (i.e., tumor and kidneys) indicating FR-mediated uptake. The majority of the activity in the tumor was retained at 24 h post-injection for both radiofolates, however, [^55^Co]Co-cm10 showed a small decrease in tumor uptake (13% ± 2% IA/g), while [^55^Co]Co-rf42 did not (15% ± 6% IA/g). The PET/CT images (presented as maximal intensity projections) confirm the biodistribution findings, highlighting the kidneys and tumors as the tissues with the highest activity uptake (Figure 5).

It is known that the in vivo behavior of a radiopharmaceutical can be altered by changing the bifunctional chelating agent, as is the case with [^55^Co]Co-cm10 and [^55^Co]Co-rf42—two compounds that differ in their respective DOTA and NODAGA chelators [24]. In the case of [^55^Co]Co-cm10, DOTA binds to Co(II) via four amino N donors and two carboxylic acid O donors resulting in a neutral [Co(N_4_O_2_)] core, while the remaining carboxylic acid O is deprotonated at physiological pH (pK_a_ = 4.04) resulting in an overall −1 charge [20,25,26]. The NODAGA-functionalized folate derivative (rf42) binds to the Co(II) core via three amino N donors and three carboxylic acid O donors, resulting in a monoanionic [Co(N_3_O_3_)]^1-^ core. It is unclear whether differences in the coordination mode between [^55^Co]Co-cm10 and [^55^Co]Co-rf42 contribute significantly to differences observed in vivo as each complex has the same overall −1 charge.

**Figure 6:**
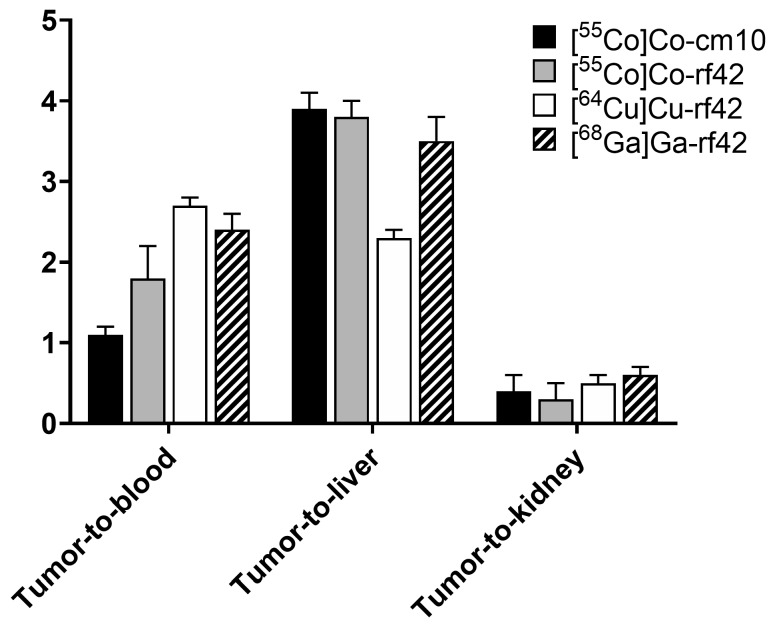
Tumor-to-organ ratios for previously reported complexes [^64^Cu]Cu-rf42 and [^68^Ga]Ga-rf42 in comparison to [^55^Co]Co-rf42 and [^55^Co]Co-cm10 [14].

In comparison to previously reported [^64^Cu]Cu-rf42 and [^68^Ga]Ga-rf42, the ^55^Co-labeled folates show some moderate differences when comparing tumor-to-tissue ratios (Figure 6) [14]. The primary difference between the previously reported ^64^Cu- and ^68^Ga-labeled radiofolates and this study (other than the choice of radiometal) is the comparatively lower effective molar activity of the ^55^Co-labeled radiofolates, which resulted in the injection of an approximately 5-fold higher amount (2.4 nmol) of folate versus the 0.5 nmol injected in previous studies. The two ^55^Co-labeled radiofolates both showed much higher tumor-to-liver ratios in comparison to [^64^Cu]Cu-rf42, but lower tumor-to-blood ratios than [^64^Cu]Cu-rf42 and [^68^Ga]Ga-rf42 at 4 h post-injection. The lowest tumor-to-kidney ratio was exhibited by [^55^Co]Co-rf42, while [^55^Co]Co-cm10 exhibited a tumor-to-kidney ratio similar to both the ^64^Cu- and ^68^Ga-labeled radiofolates. The ^55^Co-labeled compounds (17% ± 2% IA/g for [^55^Co]Co-cm10 and 17% ± 4% IA/g for [^55^Co]Co-rf42) had the highest overall tumor uptake at 4 h post-injection ([^64^Cu]Cu-rf42 = 14.5% ± 1.0% IA/g; [^68^Ga]Ga-rf42 = 12% ± 2% IA/g). However, by 24 h post-injection [^64^Cu]Cu-rf42 uptake increased to 16% ± 4% IA/g, while [^55^Co]Co-rf42 remained statistically unchanged at 15% ± 6% IA/g and [^55^Co]Co-cm10 tumor uptake, while still high, decreased slightly to 13% ± 2% IA/g.

In summary, two albumin-binding folate derivatives cm10 and rf42 were radiolabeled with the positron-emitter ^55^Co and evaluated pre-clinically. The new compounds-[^55^Co]Co-cm10 and [^55^Co]Co-rf42-were each prepared in high yield (≥95%) and exhibited excellent in vitro stability over 24 h in PBS (93%) and mouse serum (94%). The two radioconjugates exhibited almost identical in vitro characteristics, but differed when evaluated in vivo. Both compounds exhibited excellent tumor uptake (17% IA/g) at 4 h post-injection with [^55^Co]Co-cm10 exhibiting lower renal uptake (36% IA/g) and higher blood uptake (14% IA/g) in comparison to 53% IA/g and 8% IA/g, respectively, for [^55^Co]Co-rf42. The slower blood clearance rate and lower renal uptake exhibited by [^55^Co]Co-cm10 is advantageous for FR-targeting, and both cm10 and rf42 formed complexes with ^55^Co that remained stable in vivo. Therefore, it appears that the choice of chelator for ^55^Co is versatile and can be manipulated to fine-tune in vivo behavior of ^55^Co-labeled bioconjugates without sacrificing stability. 

As with all folic-acid based FR-targeting agents, the ^55^Co-labeled radiofolates cannot distinguish between the FRα and the FR beta isoform (FRβ). Expression of the FRβ on activated macrophages offers the potential for using folic-acid based agents to image inflammatory diseases (e.g., rheumatoid arthritis, osteoarthritis), however, it also presents a challenge to the use of folic acid based diagnostic agents for oncologic imaging, due to the possibility of a false positive result [27,28]. Nevertheless, the high tumor uptake, comparable tumor-to-kidney ratios and higher tumor-to-liver ratios achieved by the ^55^Co-labeled folate derivatives (in comparison to similar ^64^Cu- and ^68^Ga-labeled compounds) offer a strong case for the continued investigation of ^55^Co-labeled bioconjugates for use in nuclear medicine. 

## 3. Materials and Methods 

### 3.1. General Methods and Instrumentation

All chemicals were of trace metals grade and purchased from Fisher Scientific (Hampton, NH, USA) or Sigma Aldrich (St. Louis, MO, USA) unless otherwise specified. Glassware was acid washed overnight using 6 M HNO_3_ prior to use. Isotopically enriched nickel-58 (^58^Ni) metal (99.8% enrichment) was purchased from IsoFlex USA (San Francisco, CA, USA). All water used was deionized 18 MΩ.cm high resistivity water, purified using a Milli-Q system (EMD Millipore, Burlington, MA, USA). For separations and radiolabeling, this water was further treated by mixing 1 L of water with 50 g of Chelex 100 resin (Bio-Rad, Hercules, CA, USA) at room temperature for 1 h before filtration through a 0.2 μm sterile vacuum filter (EMD Millipore, Burlington, MA, USA). 

HPLC analyses were performed using an Agilent 1260 Infinity system (Santa Clara, CA, USA) equipped with a UV-Vis detector followed in-line by a Flow-RAM NaI detector (LabLogic, Brandon, FL, USA). A BetaBasic-18 Column (Thermo Scientific, Waltham, MA, USA) was used for analysis (150 mm × 4.6 mm, 5 μm). HPLC solvent A was HPLC-grade acetonitrile (ACN) with 0.1% HPLC-grade trifluoroacetic acid (TFA), and solvent B was water with 0.1% TFA, which was filtered through a 0.2 μm filter prior to use. The gradient was 5–80% solvent A in solvent B over 20 min with a flow rate of 1 mL/min and UV monitoring at 254 nm. Subsequent data analyses were accomplished using Laura software, Version 4.5 (LabLogic, Brandon, FL, USA). 

Radio-TLC was performed using Al-backed silica TLC plates (1 cm × 7 cm) developed with pH 4.6 citric acid buffer (reagent grade). The TLC plates were analyzed using an AR-2000 Imaging Scanner (Eckert and Ziegler, Hopkinton, MA, USA) and the accompanying WinScan 3.1 software.

### 3.2. Cobalt-55 Production and Purification

Targets were prepared by electroplating enriched ^58^Ni onto the surface of a Au coin using a previously described method [29]. The electroplating apparatus was built by the University of Alabama at Birmingham (UAB) Machine Shop and based on a published design [30]. The electroplating solution was prepared by dissolving 30–40 mg of Ni metal powder in 9 M HCl in a 20 mL beaker with heating at 100 °C. Heating was continued until complete evaporation of the HCl was achieved, wherein the remaining yellow NiCl_2_ residue was cooled and dissolved in an aqueous 30 mg/mL boric acid solution to a final Ni concentration of 0.5 M. The resulting solution was transferred to a cylindrical electroplating cell (4.5 cm × 1.8 cm I.D.) that was seated on a Teflon base with a circular opening (5 mm radius) exposing the Au-coin cathode. A rotating Pt-rod anode (84 rpm) was used, and a potential of 3.6 V was applied to the solution for 8 to 12 h, the current varied from 20–40 mA. Targets were bombarded on an ACSI TR24 Cyclotron (Richmond, BC, Canada) for 1 to 2 h with 18 MeV protons and currents of 30 or 40 μA. Targets were retrieved for processing 1 h after the end of bombardment (EOB). 

Post-bombardment target processing was performed using a modified version of a previously reported method [31]. Targets were placed in 5 mL of 9 M HCl and heated at 100 °C for 1 h to fully dissolve the Ni from the surface of the Au coin. The Ni solution was cooled and loaded onto a 5 cm × 1 cm I.D. glass column (Bio-Rad, Hercules, CA) containing 2.4 g of AG-1 × 8 resin (Bio-Rad, Hercules, CA). The column was eluted with 10 mL of 9 M HCl to collect the bulk Ni followed by two 2 mL fractions of 0.5 M HCl; and the second fraction contained the ^55^Co product. To further purify and concentrate the radioactivity, 1.5 mL of concentrated HCl was added to the 2 mL ^55^Co fraction, and the re-acidified solution was loaded onto a Bio-Rad polypropylene column (3 cm × 0.8 cm I.D.) containing 1.4 g of AG-1 × 8 resin. The column was eluted with 10 mL of 9 M HCl followed by two 1 mL fractions of 0.5 M HCl, of which the final 1 mL contained the majority of the ^55^Co product. The final fraction was evaporated to dryness by heating at 98 °C, and the radioactivity was reconstituted in 20 μL of 0.1 M HCl. 

### 3.3. Preparation of ^55^Co-labeled Radiofolates

The albumin-binding folate conjugates cm10 and rf42 were provided by Dr. C. Müller, Paul Scherrer Institute, Switzerland [13,14]. Each precursor was dissolved in an aqueous 0.5 M sodium acetate solution (pH 8) to target a concentration of 1 mg/mL to serve as stock solutions. Prior to use, the ^55^Co solution was pH adjusted with 0.5 M NH_4_OAc to achieve a pH of 5 for radiolabeling. 

For radiolabeling, 10–75 μL of cm10 or rf42 stock solution (7–40 nmol) was added to a 1.5 mL centrifuge tube. To this, 1–15 μL of ^55^Co stock solution was added 3.0–37 MBq (0.08–1 mCi) and the volume was adjusted to 10–75 µL with 0.5 M ammonium acetate (pH = 6) to obtain a final concentration of approximately 0.5–0.7 mM folate and a final reaction pH of 5.5–6.5. The mixture was heated on an Eppendorf ThermoMixer C (Hamburg, Germany) at 50 °C for 30 min. Characterization of the radiolabeled folates was performed by HPLC comparison against the non-radiolabeled precursor. Radiochemical yield (RCY) was determined via radio-TLC. 

### 3.4. In Vitro Stability Determination

The ^55^Co-labeled folate derivatives were diluted 10-fold in 10 mM PBS (pH = 7.4) or whole mouse serum (EMD Millipore, Burlington, MA) in 1.5 mL centrifuge tubes to final volumes of 100 μL. The tubes were incubated at 37 °C with vortexing at 900 rpm using an Eppendorf ThermoMixer C (Eppendorf, Hamburg, Germany) equipped with a 1.5 mL heating block. In the case of PBS stability, three replicate tubes were prepared with average final activity concentrations of 37 kBq (1 μCi) [^55^Co]Co-cm10/μL or 37 kBq (1 μCi) [^55^Co]Co-rf42/μL, and the final concentrations of precursor were 0.07 mM of cm10 or rf42. The percent of the intact complex was determined by analyzing 1 μL aliquots of each solution by radio-TLC at 1 h, 4 h and 24 h. For mouse serum stability, three replicate tubes were prepared for each time point for each complex. The final average activity concentrations were 11.1 kBq (0.3 μCi) [^55^Co]Co-cm10/μL and 7.4 kBq (0.2 μCi) [^55^Co]Co-rf42/μL and 0.02 mM final folate concentration. A control sample was prepared for each time point that contained an average of 10.4 kBq/μL of [^55^Co]CoCl_2_. At each time point, 300 μL of ACN was added to each tube to precipitate serum proteins. The tubes were centrifuged at 4500 rpm for 5 min using a benchtop microcentrifuge (Eppendorf, Hamburg, Germany) and the supernatants collected. The pellets were washed once more, and the supernatants were collected and combined with the first ACN wash. The activities in the pellets and supernatants were determined using a 2480 Wizard2 Automatic Gamma Counter (Perkin Elmer, Waltham, MA, USA) to determine the amount of activity associated with the protein pellet. To determine the percentage of intact complex, the supernatant was diluted 2-fold with water, and 100 μL of this mixture was injected into the HPLC.

### 3.5. Cell Culture

The human cervical cancer cell line KB (CCL-17) was purchased from ATCC (Manassas, VA, USA) and cells were cultured in Gibco folate-deficient RPMI 1640 growth medium (FFRPMI, Thermo Scientific, Waltham, MA, USA) supplemented with 10% fetal bovine serum, and Gibco 1% Antibiotic-Antimycotic. Cells were cultured following standard procedures and were incubated at 37 °C in a 5% CO_2_ environment and harvested using Gibco 0.25% trypsin-EDTA.

### 3.6. Cell Binding and Internalization

Cell internalization was performed similarly to a previously published procedure [14]. KB cells were harvested, washed with supplemented FFRPMI, and resuspended in supplemented FFRPMI at a final concentration of 1 × 10^5^ cells/mL for seeding. In 24 mL plates, 1 mL of the cell mixture was added to each well, and the plates were incubated overnight to allow for adhesion and growth. The following morning, the radiofolates were prepared at molar activities of 331.7 MBq/μmol (9.0 mCi/µmol) [^55^Co]Co-cm10 and 299.5 MBq/μmol (8.1 mCi/μmol) in 50 μL final volume and then diluted in supplement free FFRPMI (either with or without 1 mM folic acid) to a final volume of 1 mL/well. The plates were collected, the supernatants discarded, and 1 mL of the dilute radiofolates in media was added to each well (n = 4 for each condition). The final molar activities for each condition were, as follows—(a) 290.2 MBq/μmol (7.8 mCi/μmol) [^55^Co]Co-cm10; (b) 290.2 MBq/μmol (7.8 μCi/μmol) [^55^Co]Co-cm10 with 0.7 mM folic acid; (c) 250.4 MBq/μmol (6.7 μCi/μmol) [^55^Co]Co-rf42; (d) 250.4 MBq/μmol (6.7 mCi/μmol) [^55^Co]Co-rf42 with 0.7 mM folic acid. Following this, cells were incubated at 37 °C for 0.5 h or 2 h. Afterwards, the supernatant was removed, and cells were washed twice with 1 mL of ice cold PBS buffer followed by 1 mL of stripping buffer (0.1 M acetic acid in 0.15 M saline, pH 3) to remove FR-bound radiofolates from the cell surface [32]. The cells were lysed by addition of 1 mL of 1 M NaOH in PBS to each well. The stripping buffer and lysed cell fractions were collected separately and counted using an automatic gamma counter. 

The lysed cell fractions in NaOH were saved, and the total protein concentration for each sample was determined using a Pierce Micro BCA Protein Assay kit (Thermo Scientific, Waltham, MA, USA). The samples were vortexed vigorously prior to assaying, and were analyzed without further dilution. The results were used to normalize the measured radioactivity to the average protein content in each well.

### 3.7. In Vivo Biodistribution and PET Imaging

All animal studies were performed using a protocol approved by the Institutional Animal Care and Use Committee at the University of Alabama at Birmingham and were in compliance with national animal welfare policies and guidelines. Female athymic nude mice (Charles River, Wilmington, MA, USA), age 5 weeks, were implanted with KB cells via subcutaneous injection of 5 × 10^6^ cells in 100 μL of PBS in the right shoulder. Tumors were allowed to grow for 12 days, during which time mice were fed Teklad Folic Acid Deficient Diet (Envigo, Huntingdon, United Kingdom). 

Biodistribution and imaging studies for [^55^Co]Co-cm10 and [^55^Co]Co-rf42 were performed at 4 h and 24 h post-injection on groups of four mice. An additional cohort of mice was co-injected with a blocking dose of folic acid (100 μg), and biodistribution was performed at 4 h post-injection. At 2 h, mice were induced using 3% isoflurane and a 50 μL blood sample was taken retro-orbitally to monitor blood activity at an early time point. 

The radiofolates were prepared as described with final molar activities of 835.5 MBq/μmol (22.6 mCi/μmol) for [^55^Co]Co-cm10 and 957.6 MBq/μmol (25.9 mCi/μmol) for [^55^Co]Co-rf42 in 60 μL reaction volume. Injections (100 µL) were prepared by dilution of the radiofolates in sterile saline (pH = 7). The final [^55^Co]Co-cm10 injection contained 1,924 kBq (52 μCi) and 2.3 nmol cm10 and the final [^55^Co]Co-rf42 injection contained 2,479 kBq (64 μCi) and 2.4 nmol rf42. Blocking doses contained an additional 100 µg of folic acid and were prepared by diluting the radiofolates with 1 μg/μL folic acid in sterile saline (pH adjusted to 7). Mice were euthanized humanely by cervical dislocation following anaesthetization with 3% isoflurane, and their organs were collected, weighed and counted using an automatic gamma counter. 

Mice in the 24 h biodistribution group were imaged at 4 h and 24 h prior to biodistribution (total eight mice). PET/CT scans were performed at 4 h and 24 h on live mice using a GNEXT PET/CT (Sofie Biosciences, Dulles, VA, USA) imaging scanner. Mice were anaesthetized with 3% isoflurane at induction and 2.5% isoflurane during imaging and were kept warm via a heated imaging platform. Static whole-body PET scans were acquired for 20 min (4 h time points) and 30 min (24 h time points), and CT scans were collected for 3 min. The images were reconstructed by 3D-OSEM using the integrated GNEXT Acquisition Engine software and post-processed with VivoQuant (Invicro, Boston, MA, USA) software.

## Figures and Tables

**Table 1:** Stability of [^55^Co]Co-cm10 in PBS and mouse serum ^a^.

	1 h	4 h	24 h
PBS	97 ± 2	95 ± 1	93.4 ± 0.3
Mouse Serum	98 ± 1	97.8 ± 0.6	95 ± 1

^a^ Values reported as mean percent intact complex ± SD and normalized to percentage of intact complex at 0 h, n = 3.

**Table 2:** Stability of [^55^Co]Co-rf42 in PBS and mouse serum^a.^

	1 h	4 h	24 h
PBS	98 ± 2	95.6 ± 0.9	93 ± 3
Mouse Serum	97.5 ± 0.5	97.2 ± 0.4	94.6 ± 0.6

^a^ Values reported as mean percent intact complex ± SD and normalized to percentage of intact complex at 0 h, n = 3.

**Table 3:** Biodistribution of [^55^Co]Co-cm10 in KB tumor bearing mice ^a^.

Organ	4 h	4 h Blocking ^b^	24 h
Blood	13.8 ± 0.8	17 ± 3	2.6 ± 0.3
Heart	4.7 ± 0.6	4.2 ± 0.7	1.7 ± 0.2
Lungs	5.4 ± 0.6	7 ± 3	1.9 ± 0.3
Pancreas	2.4 ± 0.7	2.4 ± 0.7	1.4 ± 0.2
Spleen	2.0 ± 0.4	2.2 ± 0.4	1.0 ± 0.1
Stomach	0.8 ± 0.2	1.5 ± 0.1	0.34 ± 0.09
Liver	3.7 ± 0.6	3.5 ± 0.5	1.9 ± 0.3
Kidney	36 ± 7	16 ± 4	30 ± 2
Intestine	1.9 ± 0.3	2.3 ± 0.4	0.92 ± 0.08
Fat	5 ± 1	4 ± 1	3 ± 2
Skin	6 ± 2	5 ± 1	7 ± 3
Muscle	2.4 ± 0.9	2.1 ± 0.7	1.3 ± 0.3
Bone	1.8 ± 0.6	2.2 ± 0.8	0.8 ± 0.2
Brain	0.8 ± 0.1	0.6 ± 0.2	0.45 ± 0.08
Tumor	17 ± 2	8 ± 4	13 ± 2

^a^ Values reported as % IA/g ± SD, n = 4; ^b^ Co-administration of a 100 µg folic acid blocking dose.

**Table 4:** Biodistribution of [^55^Co]Co-rf42 in KB tumor bearing mice ^a^.

Organ	4 h	4 h Blocking ^b^	24 h
Blood	8 ± 3	15 ± 2	1.0 ± 0.4
Heart	2.9 ± 0.4	4 ± 1	2.0 ± 0.4
Lungs	4 ± 1	5.6 ± 0.3	1.5 ± 0.4
Pancreas	2.4 ± 0.5	2.2 ± 0.2	1.9 ± 0.3
Spleen	1.5 ± 0.3	2.1 ± 0.3	0.6 ± 0.2
Stomach	0.8 ± 0.4	1.1 ± 0.5	0.5 ± 0.2
Liver	3.8 ± 0.6	4 ± 1	2.9 ± 0.5
Kidney	53 ± 12	12 ± 4	46 ± 14
Intestine	2.2 ± 0.5	2.2 ± 0.3	0.8 ± 0.2
Fat	3.2 ± 0.9	2.7 ± 0.6	2.7 ± 0.2
Skin	8 ± 1	3.5 ± 0.8	6 ± 2
Muscle	1.6 ± 0.4	1.5 ± 0.3	1.2 ± 0.6
Bone	1.3 ± 0.2	1.7 ± 0.5	0.6 ± 0.2
Brain	0.7 ± 0.1	0.5 ± 0.1	0.4 ± 0.1
Tumor	17 ± 4	7 ± 2	15 ± 6

^a^ Values reported as % IA/g ± SD, n = 4; ^b^ Co-administration of a 100 µg folic acid blocking dose.

## Supplementary Materials

The following are available online at https://www.mdpi.com/1424-8247/12/4/166/s1, Figure S1: HPLC chromatograms of [^55^Co]Co-cm10 after incubation in mouse serum at 37 °C for 1 h, 4 h and 24 h., Figure S2: HPLC chromatograms of [^55^Co]Co-rf42 after incubation in mouse serum at 37 °C for 1 h, 4 h and 24 h.
