# Supplementary material for: New 55Co-labeled Albumin-Binding Folate Derivatives as Potential PET Agents for Folate Receptor Imaging
Source: Pharmaceuticals (Basel). 2019 Nov 8;12(4):166. doi: 10.3390/ph12040166 (PMC6958329; doi:10.3390/ph12040166)
Supplement: Supplementary file 1 [file pharmaceuticals-12-00166-s001.pdf]

Article

New ^{55}Co-labeled Albumin-binding Folate Derivatives as Potential PET Agents for Folate Receptor Imaging

Lauren L. Radford[1], Solana Fernandez[1], Rebecca Beacham[1], Retta El Sayed[1], Renata Farkas[2], Martina Benešová[2,3], Cristina Müller[2,3], Suzanne E. Lapi[1,4*]

[1] Department of Radiology, University of Alabama at Birmingham, Birmingham, AL 35233, United States; lauren.radford@curiumpharma.com (L.L.R.); sfernandez@uabmc.edu (S.F.); beachambecca@gmail.com (R.B.); relsay2@emory.edu (R.E.S.)

[2] Center for Radiopharmaceutical Sciences ETH-PSI-USZ, Paul Scherrer Institute, 5232 Villigen-PSI, Switzerland; dr.renata.farkas@gmail.com (R.F.); m.benesova@dkfz-heidelberg.de (M.B.); cristina.mueller@psi.ch (C.M.)

[3] Department of Chemistry and Applied Biosciences, ETH Zurich, 8093 Zurich, Switzerland

[4] Department of Chemistry, University of Alabama at Birmingham, Birmingham, AL 35205, United States

* Correspondence: lapi@uab.edu

Supplemental data for in vitro stability in mouse serum

Representative chromatograms of [^{55}Co]Co-cm10 and [^{55}Co]Co-rf42 after incubation at 37 °C in mouse serum over a period of 24 h.

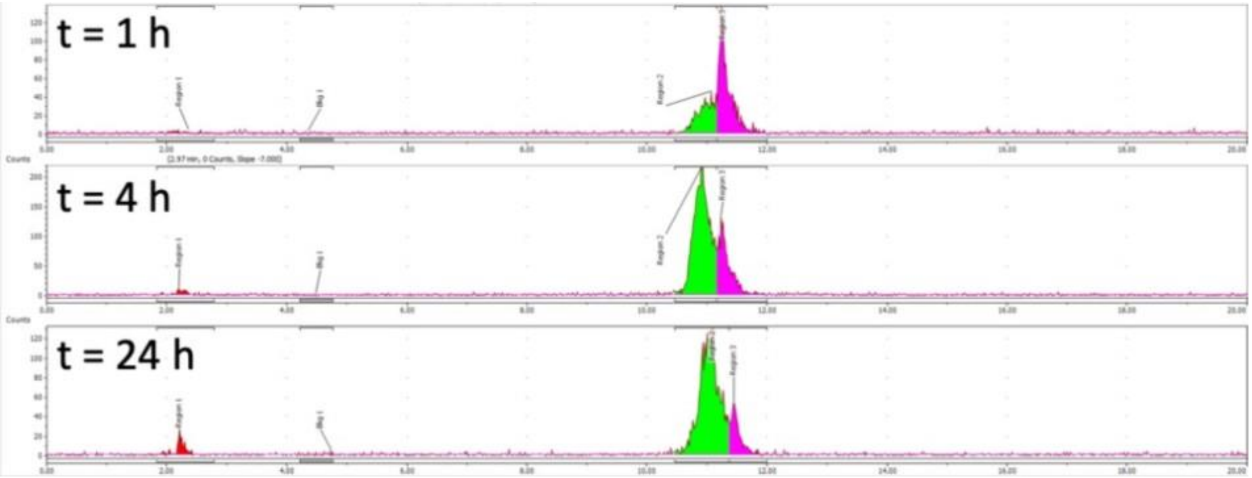

Figure S1. HPLC chromatograms of [^{55}Co]Co-cm10 after incubation in mouse serum at 37 °C for 1 h, 4 h and 24 h. The area highlighted in pink is the retention time of the original complex (t_R = 11.3 min). The area highlighted in green shows the ingrowth of a new peak (t_R = 11 min). Free [^{55}Co]$CoCl_2$ elutes at the beginning of the chromatogram (t_R = 2.2 min).

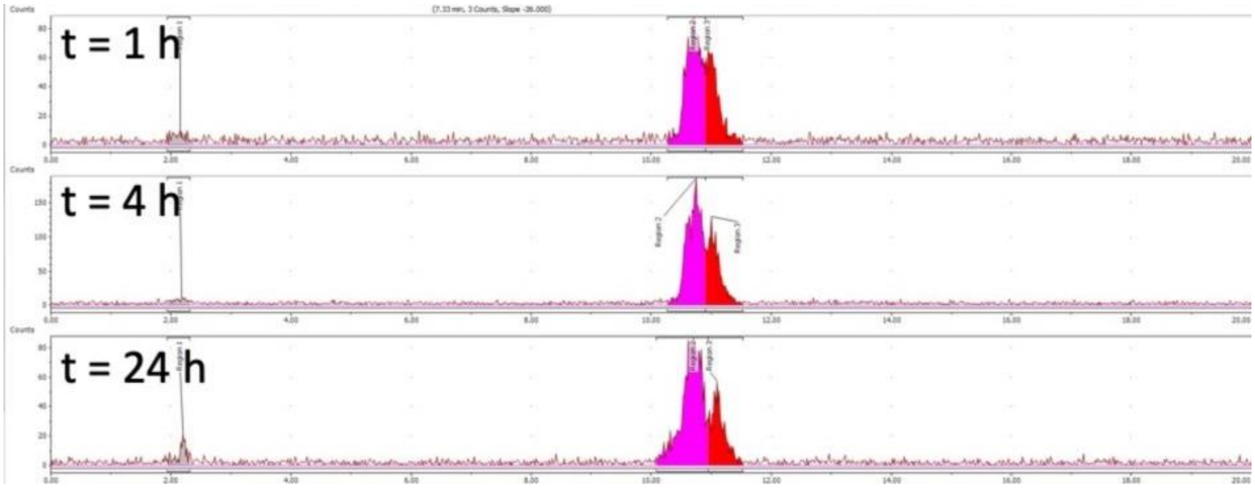

Figure S2. HPLC chromatograms of [^{55}Co]Co-rf42 after incubation in mouse serum at 37 °C for 1 h, 4 h and 24 h. The area highlighted in pink is the retention time of the original complex (t_R = 10.7 min). The area highlighted in red shows the ingrowth of a new peak (t_R = 10.9 min). Free [^{55}Co]$CoCl_2$ elutes at the beginning of the chromatogram (t_R = 2.2 min).
